# Hereditary and non-hereditary branches of family eligible for BRCA test: cancers in other sites

**DOI:** 10.1186/s13053-017-0067-8

**Published:** 2017-05-25

**Authors:** M. Digennaro, D. Sambiasi, S. Tommasi, B. Pilato, S. Diotaiuti, A. Kardhashi, G. Trojano, A. Tufaro, A. V. Paradiso

**Affiliations:** 1 Centro Studi Tumori Eredo-familiari. Istituto Tumori G Paolo II,IRCCS, 70124 Bari, Italy; 2 Laboratorio Genetica Molecolare; Istituto Tumori G Paolo II, IRCCS, 70124 Bari, Italy; 3 UO Senologia Tumori. Istituto Tumori G Paolo II,IRCCS, 70124 Bari, Italy; 4 UO Ginecologia Oncologica, Istituto Tumori G Paolo II, IRCCS, 70124 Bari, Italy; 5 ASST Fatebene Fratelli, Milan, Italy; 6 Biobanca Istituzionale, Istituto Tumori G Paolo II, IRCCS, 70124 Bari, Italy; 7 Centro Studi Tumori Eredo-Familiari, Istituto Tumori G Paolo II, IRCCS, Via O. Flacco, 65, 70124 Bari, Italy

**Keywords:** Breast/ovarian cancer, Hereditary syndrome, Cancer in other sites, Hereditary/non hereditary branch

## Abstract

**Background:**

The analysis of relationships of BRCA alterations with cancer at sites other than breast/ovary may provide innovative information concerning BRCA pathogenic role and support additional clinical decisions. Aim of this study is to compare presence of cancers in other sites in members of hereditary (H) and not-hereditary (nH) branches of families of patients eligible to BRCA test.

**Methods:**

We retrospectively analyzed the incidence of cancer in other sites in members of 136 families eligible for hereditary breast/ovarian cancer genetic counseling at Centro Studi Tumori Eredo-familiari of our Institute; we compared the frequency of other cancer types in 1156 members of the H-branch with respect to 1062 members of nH-Branch. The families belonging to a proband case and with informative members in at least three generation entered the present study.

**Results:**

The frequency of other Cancers in members of H-branch was significantly higher than that in members of nH-branch (161 vs 75 cancers; *p* < 0.0001). In specific, members of H-branch had a significantly higher probability to have more lung cancer (38 vs 9;*p* < 0.0006), kidney cancer (23 vs 5;*p* < 0.0005), liver cancer (13 vs 3;*p* < 0.02) and larynx cancer (14 vs 4;*p* < 0.03). Interestingly, to belong to H-branch resulted significantly associated with a higher probability of lung cancer (OR 4.5; 2.15–9.38 95%C.I.), liver cancer (OR: 4.02; 1.14–14.15 95% C.I.) and larynx cancer (OR:3.4; 1.12–10.39 95%C.I.) independently from Gender and Age.

**Conclusions:**

Members belonging to the H-branch of families of patients eligible to BRCA test have a higher risk of tumors in lung, larynx and liver. Clinicians should consider the increased risk for these cancers to activate prevention/early diagnosis practices in members of families with breast/ovarian familial cancer syndrome.

## Background

Germ line mutations in BRCA1 and BRCA2 genes generally account for hereditary breast/ovarian cancer and, when found, permit to predict the life-time risk for these cancers [1]. However, it has already been clearly demonstrated that this hereditary cancer syndrome implies a higher risk of malignancy also in other than breast/ovarian sites [2, 3]. In specific, BRCA alterations have been reported to be associated with cancers in colon, pancreas, prostate, stomach, uterus, liver and melanomas [4].

The evidence of an association of BRCA1/2 alterations with cancers in sites other than breast and ovary is of potential great relevance for several reasons. First of all, the demonstration that these gene alterations are important for the development of a wider spectrum of cancers should stimulate innovative in vitro/in vivo organ-specific cancerogenesis studies [5, 6]; furthermore, the increased incidence of cancers to other sites may reflect yet unknown non-BRCA related diseases potentially supporting the possibility to utilize the information for a more precise genetic risk calculation [7]; lastly, the acquisition of new information on other associated cancers could support the adoption of specific clinical prevention/early diagnosis practices in members of families fulfilling “ad hoc” criteria [8, 9].

However, information on other BRCA-related cancers have been obtained till now from observational studies considering series of mutation carriers only [10], large unselected populations [11] or country wide registries [12] thus producing not homogeneous information on cancer risk for different organs; the heterogeneity of the evidences supported the idea that other genetic alterations or other non-genetic characteristics (i.e., environmental exposure, voluptuary habit) could play a significant role in modulating pathogenic BRCA impact [13]. In spite of these hypothesis, no studies have been already published verifying the possibility of an association of other cancer types with hereditary breast/ovarian cancer syndrome (HBOC) by a direct comparison of frequency of various cancers in Hereditary (H) and not-Hereditary (nH) branches of the family belonging to the same proband BRCA patient. In our opinion, this model should represent,, the ideal one to study hereditary-familial cancer frequency in subjects living in similar environmental-voluptuary-lifestyle conditions. Aim of the present study is to analyze the risk for cancers in other sites associated with HBOC in a mono-institutional consecutive Caucasian series of subjects leaving in Apulia Region and belonging to H or nH branches of families of the same proband patient.

## Methods

All the families that had access for a genetic counseling for HBOC syndrome to the Centro Studi Tumori Eredo-Familiari of Istituto Tumori G Paolo II of Bari from 2004 to 2008 were considered for eligibility to the present study. One hundred fifty five out of 320 considered families met the criteria to enter the study: 1) availability of the proband case; 2) BRCAPRO risk for genetic alteration of the proband patient >20%; 3) oncologic information of relatives available at least for three generations; 4) H branch clearly and unequivocally identifiable through analysis of family trees according to the presence of breast/ovarian cancer in its members. Twenty three out 155 families were excluded from further analysis because familiarity was not enough clearly attributable to only one specific family branch. Overall, oncologic information on 2218 subjects from 132 families were available with 1156 members belonging to H branch and 1062 to nH branches. Of 132 families entering the study, in 88 cases the H branch was identified in the maternal lineage whereas in 44 cases in paternal lineage. Proband patients from 103 families were screened for germ line mutations in BRCA1 and BRCA2 genes while the genetic test was not performed in the remaining 29 cases. A pathogenic mutation in BRCA 1 or 2 genes was found in 22 and 12% of analyzed cases, respectively. All patients candidate to BRCA1 and BRCA2 mutation analysis signed an informed consent. Oncologic information were collected within an “ad hoc” interview drawn by expert personnel belonging to the Centro Studi Tumori Eredo-familiari. A questionnaire, as previously reported [14], has been preliminarily submitted to proband patients to evaluate the attitude to provide reliable information involving the family. Only information on oncologic history of the family considered reliable were retained for further analysis.

### Genetic analysis

Molecular analysis of BRCA 1–2 genes was conducted on DNA extracted from blood lymphocytes of proband patients; all exons of BRCA1 and 2 genes have been analysed by direct sequencing as previously described [15].

### Statistical analyses

The frequency of different cancers in members of H and nH branches of the families was compared by two-tailed chi-square test, with Yates correction. Logistic regression analysis, with frequency of cancer in different sites as dependent variable and age at diagnosis, sex and belonging to H or nH branches included in the model, was performed. Results have been considered statistically significant for *p* values < 0.05. Statistical analyses were carried out with the SPSS statistical software (SPSS, inc, Chicago, IL).

## Results

Of the 132 families taking part in the study, 1156 and 1062 members belonged to the H branch and nH branches, respectively. Mean number of members of the families were similar for H and nH branches (8.7 vs 8.1 members, respectively) and for families carrying or not carrying a BRCA mutation (7.8 vs 8.5 members, respectively). In Table 1, main oncologic characteristics of members of the two different family branches are reported. As expected, frequency of total cancers, breast cancers and ovarian cancers was significantly higher in H branch with respect to nH-branch. In particular, cancer in sites other than breast and ovary occurred in 13.9% of members belonging to the H branch with respect to 7.0% of the nH branch (*p* < 0.0001). Worth of note, the diagnosis of breast/ovarian cancers in H branch was confirmed to appear in subjects significantly younger.

**Table 1:** CHARACTERISTICS OF THE SERIES: Cancers in Hereditary and Non-Hereditary branches of members of families with hereditary breast/ovarian cancer syndrome

Characteristics	Hereditary Branch	Non-Hereditary Branch	*P* value (chi-square)
Relatives (number)	1156	1062	
Mean Age (years)	65.9	69.1	
Range (years)	(1–102)	(3–98)	
Number of cancers	376	90	<0.0001
Breast cancer (number)	182	13	<0.0001
Mean Age at Diagnosis	54.4	63.8	
Ovarian cancer (number)	33	2	<0.0001
Mean Age at Diagnosis	52.1	59.5	
Other Cancers (number)	161	75	<0.0001
Mean Age at Diagnosis	65.2	65.4	

In Table 2, the frequency of different types of cancers is reported. Again, significant differences became evident between the two family branches; a higher frequency in H with respect to nH branch was evident for lung cancer (38 vs 9 cases respectively; *p* < 0.0003), kidney cancer (23 vs 5 cases, respectively; *p* < 0.0005), liver cancer (13 vs 3 cases, respectively; *p* < 0.02), larynx cancer (14 vs 4 cases, respectively; *p* < 0.03). When analysis was selectively conducted in the 34 families carrying a BRCA 1/2 mutation, we confirmed a significant difference for lung and kidney cancer in the H branch but only a trend for more liver and larynx cancers. Lastly, a logistic regression analysis has been performed to check which variables were independently associated with cancers to different sites (Table 3). Age, gender and the two family branches were included in the model. Overall, we confirmed a higher relative risk (OR: 4.03; 95% CI 1.63–11.35) to have a cancer in other sites in members of H branch and this independently from age and gender characteristics. Moreover, a significant independent association was demonstrated for H branch (OR: 3.4, 95% CI 1.12–10.39) and gender (female gender OR 0.36; 95%CI:0.13–1–0;) on larynx cancer; for gender (female gender: OR 0.02; 95%CI:0.003–0.14) and H branch (OR 4.5;, 95% CI 2.15–9.38) on lung cancer; for H branch with risk of liver cancer (H branch OR 4.02, 95% CI 1.14–14.15).

**Table 2:** Other cancers in subjects belonging to Hereditary (*n* = 1156) and not Hereditary (*n* = 1062) branches of families with familial ovarian/breast cancer syndrome

Cancer site	Number of cancers in branches		*P* value^a^
	Hereditary	Not Hereditary	
Breast	182	13	0.0001
Ovary	33	2	0.0001
Lung	38	9	0.0003
Kidney	23	5	0.0005
Colon	19	16	n.s.^b^
Prostate	18	10	n.s.
Larynx	14	4	0.03
Liver	13	3	0.02
Gastric	12	9	n.s.
Leukemia	11	12	n.s.
Pancreas	6	1	n.s.
Bladder	4	4	n.s.
Melanoma	3	2	n.s.
Total	376/1156	90/1062	0.0001

^a^Chi square with Yates correction

^b^not significant

**Table 3:** Logistic regression Analysis with age, gender, BRCAPRO value and the belonging to H or nH branch with respect to probability to have other cancers among relatives

Neoplasia	Indipendent variable	Relative Risk	95% CI	*P* value
Larynx	Gender F	0.36	0.13–1.01	0.05
	H Branch	3.4	1.12–10.39	0.03
Lung	Gender F	0.02	0.003–0.14	0.000
	H Branch	4.5	2.15–9.38	0.000
Liver	H Branch	4.02	1.14–14.15	0.03
Gastric	Gender F	0.31	0.11–0.84	0.02
Other	H Branch	4.3	1.63–11.35	0.003

## Discussion

The knowledge of incidence of cancer to other sites in families with familial-hereditary breast/ovarian cancers has several potential scientific and clinical implications but information available on this argument was frequently controversial [10–12]. The possibility to compare oncologic information from H and nH branches belonging to the same family represents, for sure, one of the optimal ways to approach the problem of analysis of other cancer sites associated with this hereditary syndrome. This is the first study conducting such analysis on a mono-institutional consecutive series of Caucasian subjects living in an homogeneous geographical area. Our series of patients included in the study presented comparable frequency and age of onset of breast/ovarian cancers with respect to what already reported [1–16]. One of our most interesting results concerned the incidence of lung cancer in H branches of families at high risk for hereditary breast/ovarian cancer. In fact, we found a significantly higher number of lung cancers in members belonging to H branch (*p* < 0.0003) and, even more important, we confirmed that this higher risk (O.R.4.5; *p* < 0.000) was independent from gender. This data has been reported before [17] but not confirmed by other authors [2, 3]. The interest for BRCA role in lung cancer is increasing, recently; the role of this gene has been considered as prognostic factor [18] and recently reported as predictive biomarker of chemo-sensitivity to PARP-inhibitors [19]. Our results suggest not to underestimate the presence of lung cancers in families eligible for BRCA mutation test and the possibility to activate prevention/early diagnosis programs at least in families with specific familial feature. Recent biological evidences suggest that lung tumors could be characterized by inactivated BRCA 1/2 functional pathway due to promoter hyper-methylation [20].

The same molecular mechanisms seem common to kidney cancer [20] but alterations in CHECK2 and other genes have been also reported [21]. For kidney cancer, several genetic hereditary syndromes have been already described (von hippel landau syndrome, birt-hogg-dubè, hereditary leiomyomatosis) suggesting that a genetic high instability could basically characterize this cancer.

In our series, larynx cancer was also confirmed at univariate and multivariate analysis to be significantly associated with HBOC syndrome: to belong to the H branch of these families increases 3,4 times the risk to have a cancer to this organ while female gender resulted a protective factor. This information was already reported by the Breast Cancer Linkage Consortium [22] in BRCA2 carriers with a large analysis conducted in SEER database.

Lastly, liver cancer showed a 4.02 higher risk in members of H branch of these families. The large involvement of this organ in hormone metabolism leads to the reasonable hypothesis of its involvement in hereditary cancer syndromes in which hormones play a major role like those involving breast and ovarian cancer [23].

## Conclusion

Whne considering the results of our study, some potential limitations have to be taken into consideration; a possible recruitment bias could be related to the a priori selection of families according to BRCAPRO test which utilizes age of patients and number of subjects with cancer for its calculation; furthermore the limited number of families included in the study and, in particular, the small number of families carrying a BRCA mutation, clearly requires the confirmation of our results in larger series of cases.

The innovative aspects of our study could be summarized as follows: a) for the first time the frequency of other cancers were analyzed in the different family branches of patients with familial-hereditary syndrome; b) we confirm the H branch at significant and independent higher risk for other cancers; c) larynx cancer, lung cancer and liver cancer resulted at higher incidence in H branch and this independently from age and gender factors.

The association of various cancer types with HBOC syndrome should solicit further studies to verify the possibility to include other cancers in prevention practices and, even more interesting, to reinforce the attempt already ongoing [24, 25] to treat those tumors with PARP-inhibitors. In order to confirm this hypothesis, we are continuing our research by studying somatic BRCA status and DNA-repair pathway characteristics of tumors of patients belonging to high risk families for HBOC syndrome.

## Abbreviations

BRCA: Breast Related Cancer Antigen
H: Hereditary
NH: Not- Hereditary
